# Mechanisms Underlying Potential Therapeutic Approaches for COVID-19

**DOI:** 10.3389/fimmu.2020.01841

**Published:** 2020-07-21

**Authors:** Abdelouaheb Benani, Sanae Ben Mkaddem

**Affiliations:** ^1^Unité de Biologie Moléculaire, Institut Pasteur du Maroc, Casablanca, Morocco; ^2^U978 Institut National de la Santé et de la Recherche Médicale, Bobigny, France; ^3^UFR SMBH, Université Sorbonne Paris Nord, Bobigny, France

**Keywords:** SARS-CoV-2, COVID-19, immune therapy, monoclonal antibody, respiratory distress, cytokine treatment

## Abstract

Coronavirus disease (COVID-19) is caused by severe acute respiratory syndrome coronavirus 2 (SARS-CoV-2), which is a betacoronavirus, and is associated with cytokine storm inflammation and lung injury, leading to respiratory distress. The transmission of the virus is mediated by human contact. To control and prevent the spread of this virus, the majority of people worldwide are facing quarantine; patients are being subjected to non-specific treatments under isolation. To prevent and stop the COVID-19 pandemic, several clinical trials are in the pipeline. The current clinical trials either target the intracellular replication and spread of the virus or the cytokine storm inflammation seen in COVID-19 cases during the later stages of the disease. Since both targeting strategies are different, the window drug administration plays a crucial role in the efficacy of the treatment. Here, we review the mechanism underlying SARS-CoV-2 cell infection and potential future therapeutic approaches.

## Introduction

The members of the Coronaviridae family cause mild respiratory disease, and infection with these viruses can be transmitted between humans (1). Severe acute respiratory syndrome coronavirus (SARS-CoV) is transmitted from animals to humans, leading to severe respiratory diseases in individuals (2). SARS was discovered in Guangdong Province, China, in 2002 (3). Chinese bats serve as the natural reservoir hosts of SARS-CoV-2 (4). The human transmission of SARS-CoV requires intermediate hosts, such as animal food sources, including pangolin and cats (5). No specific antivirals or effective vaccines are available to treat or prevent SARS. In 2002 and 2003, the SARS pandemic was controlled by travel restrictions and patient isolation.

Recently, a new virus strain from the same virus family was discovered in Wuhan, Hubei Province, China, that causes coronavirus disease 19 (COVID-19) (6). It has been suggested that the human transmission of this strain was linked to the Hunan seafood market. The infection is very contagious and results in the development of the disease and fatalities (7). SARS-CoV-2 is closely related to SARS-CoV, and COVID-19 has been described as a new lung disease (8). Infections have also been detected in several countries globally and are linked to international travel. Elucidating the mechanisms through which the virus gains entry into target cells and how this process can be inhibited would allow the development of new therapeutics or vaccines to rapidly curb the ongoing pandemic. A significant number of clinical trials have been started to explore potential therapeutic strategies for COVID-19 to identify as quickly as possible high-quality efficient treatments to stop the ongoing pandemic. Here, we present a brief overview of the SARS-CoV infection mechanism and potential strategies to prevent virus entry along with the effects of infection, such as inflammatory cytokine storms, on lung injury. We discuss some published data and the mechanism of the ongoing clinical trials.

## Mechanism of SARS-CoV Cell Infection

Basically, the entry of coronavirus is mediated by the interaction of cellular receptor proteins and the S1 unit of the viral spike (S) protein, which, in turn, promotes viral attachment to the target cell surface. Furthermore, viral attachment requires cellular proteases to prime the S protein, which entails its cleavage at the S1/S2 and S2' sites, resulting in the fusion of the viral and cellular membranes. It has been shown that the S protein from SARS interacts with angiotensin-converting enzyme 2 (ACE2) as its receptor and uses the cellular serine protease TMPRSS2 to prime the S protein (9, 10). Additionally, it has been demonstrated that the SARS-S/ACE2 interaction favors the spread of the virus, leading to severe acute respiratory syndrome (11). ACE and ACE2 have high homology with metalloproteases that play a role in the renin-angiotensin system (RAS) to maintain blood pressure homeostasis. The renin protease cleaves angiotensinogen to generate angiotensin I (Ang I). The two C-terminal amino acids of Ang I are cleaved by ACE to generate angiotensin II (Ang II), whereas ACE2 cleaves Ang II. Ang II acts specifically through Ang II receptor type 1 (AT1R) and Ang II receptor type 2 (AT2R) (12, 13). ACE also degrades additional substrates such as bradykinin or apelin (14). ACE2 has been identified as the key determinant of SARS-CoV transmissibility (15). The SARS-S and SARS-2-S proteins have 76% amino acid homology. However, it is not yet clear whether SARS-2-S and SARS-S use ACE2 and TMPRSS2 for host cell binding. A recent study demonstrated that SARS-CoV-2 uses the same ACE2 receptor as SARS-CoV to enter the target cell and also uses the same cellular protease, the serine protease TMPRSS2, to prime the S protein. The study also suggested a treatment strategy based on the inhibition of S protein priming by targeting TMPRSS2 to block entry. Moreover, the study showed that sera from convalescent SARS patients cross-neutralized the S protein to block SARS-2 entry (3).

## COVID-19 and Cytokine Storm Syndrome

The antiviral response is mediated by both innate and acquired immunity, which recognize pathogen-associated molecular patterns (PAMPs) and the antigen-specific adaptive immune response. The viral response is based on the release of inflammatory mediators (cytokines, chemokines, leukotrienes, proteases, and reactive oxygen species) and on the clearance of virus through internalization and killing of the virus. Cell responses are in many ways controlled by the balance between antagonistic signals, which may affect the immune response to pathogens. The resulting balance is of great importance to prevent damage to tissues through immunopathology and to ensure the return of activated cells to a resting state. However, exaggerated and excessive synthesis of cytokines can lead to an acute, severe systemic inflammatory response known as a “cytokine storm” and cause severe damage to multiple organs (16). The cytokine profile of COVID-19 patients with differences in disease severity has been investigated, and a subset of patients with severe COVID-19 develop profound inflammation and multiorgan dysfunction that is consistent with a “cytokine storm.” Recently, a large panel of cytokines (IFN-γ, TNF-α, IL-2, IL-4, IL-6, and IL-10) and C-reactive protein (CRP) have been analyzed and compared with serum samples from a control group and from COVID-19 patients. The values for cytokines and CRP were significantly higher in patients with COVID-19 than those in healthy controls. However, using univariate logistic regression analysis, only two cytokines, IL-6 and IL-10, were found to be predictive of disease severity, suggesting that a higher level of cytokine storm is associated with severe disease development. Improving the understanding of hypercytokinemia (i.e., IL-6 levels from 100 to 5,000 pg/mL) and immune dysregulation associated with COVID-19 is urgent. Investigations of different potential therapeutic strategies for COVID-19 cytokine storm syndrome are ongoing that use corticosteroids, IL-6 blockade and IL-1 inhibition (17). A series of clinical trials of IL-6 inhibitors such as tocilizumab, sarilumab and siltuximab are also underway (see Table 1).

**Table 1:** Current therapeutic drugs used to treat COVID-19.

**Drug**	**Description and mechanism of action**	**References**
**Inhibitors of the cellular entry of SARS-CoV-2**		
Chloroquine and Hydroxychloroquine (Quensyl^TM^, Plaquenil^TM^, Hydroquin^TM^, Dolquine^TM^, Quinoric^TM^)	• Antimalarial; they have been used for decades for the prophylaxis and treatment of malaria and for various autoimmune diseases • Inhibit the terminal phosphorylation of ACE2 and elevate the pH in endosomes. • Chloroquine can inhibit the entry of SARS-CoV-2 and prevent virus-cell fusion by interfering with glycosylation of the ACE2 receptor and its binding with the spike protein, suggesting that chloroquine treatment might be more effective in the early stage of infection before COVID-19 reduces ACE2 expression and activity. • Hydroxychloroquine exhibits an anti-inflammatory effect on Th17-related cytokines (IL-6, IL-17, and IL-22) in healthy individuals and systemic lupus erythematosus and rheumatoid arthritis patients.	(18–21)
Camostat mesylate (Foipan^TM^)	• Developed decades ago for the treatment of oral squamous cell carcinoma, dystrophic epidermolysis, exocrine pancreatic enzyme inhibition, and chronic pancreatitis • TMPRSS2 protease activity as a synthetic serine protease inhibitor. In a clinical trial investigating the effects of camostat mesylate against dyspepsia associated with non-alcoholic mild pancreatic disease, 95 patients received 200 mg camostat mesylate three times daily for 2 weeks and showed only mild side effects and no severe adverse effects.	(22–24)
Nafamostat mesylate (Buipel^TM^)	• Approved in Japan for the treatment of acute pancreatitis, disseminated intravascular coagulation and for anticoagulation in extracorporeal circulation • TMPRSS2 protease activity: clinically proven as a synthetic serine protease inhibitor. Nafamostat mesylate has been shown to inhibit MERS-CoV S protein-mediated viral membrane fusion with TMPRSS2-expressing lung Calu-3 host cells by inhibiting TMPRSS2 protease activity. It may also inhibit the cellular entry of SARS-CoV-2. In cell culture experiments with simian Vero E6 cells infected with SARS-CoV-2, Nafamostat mesylate was shown to inhibit SARS-CoV-2 infection at an EC_50_ of 22.50 μM.	(19, 25, 26)
**Monoclonal antibodies targeting SARS-CoV entry**		
80R, F26G19, m396, CR3014, CR3022, F26G18, m396, 201, S230	• Binds to the conformational epitope on the S1 fragment of SARS-CoV or to the amino acid residues with high affinity on the S1 fragment of SARS-CoV. • Blocks the interaction of the S1 subunit protein with the cellular receptor ACE2	(27–32)
**Inhibitors of the replication, membrane fusion, and assembly of SARS-CoV-2**		
Remdesivir	• A novel small-molecule adenine nucleotide analog antiviral drug synthesized and developed by Gilead Sciences in 2017 that has shown efficacy against Ebola virus in rhesus monkeys. It displays antiviral activity against other single-stranded RNA viruses, including filoviruses, pneumoviruses, paramyxoviruses, and the coronaviruses MERS-CoV and SARS-CoV. • It results in the delayed chain cessation of nascent viral RNA. It potently blocks SARS-CoV-2 infection at a low range of micromolar concentrations and has a high selectivity index with an EC50 of 0.77 μM and a CC50 > 100 μM. It acts early in infection and is metabolized into its active form GS-441524, which is an adenine nucleotide analog that interferes with the activity of viral RNA polymerase and that promotes the evasion of proofreading by viral exoribonuclease, leading to the inhibition of viral RNA synthesis.	(19, 33, 34)
Lopinavir/ritonavir (Kaletra^TM^)	• Lopinavir was developed in 1998 to circumvent HIV resistance toward the protease inhibitor ritonavir. The combination of lopinavir and ritonavir was first established as an effective oral drug for the treatment of HIV-infected individuals when used in combination with other antiretroviral agents. • Lopinavir-ritonavir administration significantly decreased coronavirus titres, and low or no coronavirus titres were observed in the follow-up study. Another study investigated lopinavir in patients with COVID-19 receiving either lopinavir-ritonavir 400 mg/100 mg orally twice daily plus the standard of care or the standard of care alone.	(35)
Umifenovir (Arbidol^TM^)	• A small indole-derivate molecule licensed for oral prophylaxis and treatment of infections with influenza A and B viruses and other respiratory viruses that has been demonstrated to inhibit *in vitro* infection with globally prevalent pathogenic viruses, including the hepatitis C virus, hepatitis B virus, Ebola virus, Lassa virus, human herpesvirus, poliovirus, and vesicular stomatitis virus. • Prevents viral host cell entry by inhibiting membrane fusion of the viral envelope and the host cell cytoplasmic membrane via inhibition of clathrin-mediated endocytosis, thereby preventing virus infection.	(36)
Favipiravir (Avigan^TM^)	• An oral pyrazinecarboxamide derivative and guanine analog. • Selectively and potently inhibits the RNA-dependent RNA polymerase (RdRP) of RNA viruses (influenza A virus, flavi-, alpha-, filo-, bunya-, arena-, and noroviruses as well as West Nile virus, yellow fever virus, foot-and-mouth-disease virus, Ebola virus and Lassa virus) and induces lethal RNA transversion mutations, thereby producing a nonviable virus phenotype. A study showed favipiravir has efficacy in Vero E6 cells infected with SARS-CoV-2 with an EC50 of 61.88 μM and a CC50 over 400 μM.	(19)
**Anti-cytokines and chemokines**		
TocilizumabSarilumabSiltuximab	• Anti-IL-6 receptor is a human immunoglobulin G1 monoclonal antibody (mAb) that binds specifically to both soluble and membrane-bound interleukin-6 receptors (IL-6Rs) • Blocks the interaction between the cytokine and its receptor, avoiding the amplification of inflammation associated with lung injury that leads to respiratory distress.	(37, 38)
**Supporting agents**		
Azithromycin	• An antibiotic that can be used for different types of bacterial infections, such as respiratory and skin infections and sexually transmitted diseases. It has been proven to be active against the Zika and Ebola viruses and to prevent severe respiratory tract infections when used to treat patients suffering from viral infection. It has been used as an adjunctive therapy to provide antibacterial coverage and exerts potential immunomodulatory and anti-inflammatory effects in the treatment of some viral respiratory tract infections (e.g., influenza). • Prevents the growth of bacteria by interfering with bacterial protein synthesis. It binds to the 50S subunit of the bacterial ribosome, thus inhibiting the translation of mRNA. In COVID-19 patients, Gautret et al. reported 100% viral clearance based on nasopharyngeal swabs in six patients who were co-treated with hydroxychloroquine and azithromycin. However, Molina et al. reported findings that contrasted with those reported by Gautret. Based on those results, the data presented to date are insufficient to evaluate the possible clinical benefits of azithromycin in patients with COVID-19	(39, 40)
Corticosteroids	• A potent anti-inflammatory and anti-fibrotic drug. • Low doses of methylprednisolone prevent extended cytokine release and may accelerate the resolution of pulmonary and systemic inflammation in pneumonia. Recently, many medical researchers have stated that corticosteroids may improve the dysregulated immune response caused by sepsis (a possible complication of infection with COVID-19) and increase the blood pressure when it is low. In a retrospective cohort study, 201 patients with confirmed COVID-19 who developed ARDS were treated with methylprednisolone (1–2 mg/kg daily via IV for 5–7 days), and the results showed that treatment with methylprednisolone may be beneficial for patients who develop ARDS in terms of the reduction of the risk of death.	(41)

## Coagulation and Fibrinolysis in COVID-19

The host defense against viral infection activates the coagulation cascade to limit the spread of pathogens. During the first phase of infection, an adaptive haemostatic response occurs that is associated with the activation of a systemic inflammatory response, which is characterized by an increase in inflammatory activity and thrombin and fibrinogen generation. The increase in cytokine production during virus infection induces additional procoagulant effects, such as the expression of tissue factors that are major initiators of coagulation activation. Moreover, other factors, such as DAMPs and neutrophil extracellular traps, may also contribute to the procoagulant profile in COVID-19. During pulmonary infection, the measurement of coagulation and fibrinolysis factors in bronchoalveolar lavage fluid has demonstrated an increase in thrombin generation, an insufficient balance in physiologic anticoagulation, and the suppression of fibrinolysis, mediating the pathogenesis of respiratory distress. Endothelial injury of the pulmonary capillary is also caused by vascular endothelial damage. SARS-CoV-2 infects endothelial cells through the ACE2 receptor, and viral spread and rapid viral replication leads to massive endothelial cell apoptosis and inhibits the anticoagulant function of the vascular lumen. Moreover, endothelial dysfunction contributes to procoagulant changes in COVID-19 (42).

Platelets play a dual role; they contribute to haemostasis but also to inflammation and the host defense response, especially during lung infection. Recently, many cases of thrombocytopenia have been observed in COVID-19 patients, and the baseline platelet levels and changes were associated with subsequent mortality. However, the mechanism of SARS-CoV-2 involved in thrombocytopenia is not yet clear (43). One of the possibilities is that lung tissue injury could cause platelet activation and aggregation, and thrombi formation at the site of the injury may lead to the consumption of platelets and megakaryocytes. In addition, SARS-CoV-2 induces increases in D-dimer and fibrinogen and further increases the consumption of platelets in damaged lungs.

## Potential Therapeutic Approaches

### Monoclonal Antibodies Targeting SARS-CoV Entry

The spike proteins of SARS-CoV-2 play a major role in the interaction between the virus and the ACE2 receptor expressed by the host cell. The binding of the spike protein to ACE2 leads to membrane fusion and the initiation of the viral life cycle. To inhibit SARS-CoV-2 binding to ACE2, several neutralizing monoclonal antibodies (MAbs) targeting the spike protein of SARS-CoV-2 have been developed. Among them, the 80R MAb binds to the S1 fragment of SARS-CoV at the conformational epitope (amino acid residues 426–492) and blocks the binding of the viral S1 subunit to the ACE2 receptor, thereby preventing the entry and spread of the virus (44). These findings have been demonstrated by *in vitro* and *in vivo* studies (27, 45). Other MAbs targeting different epitopes of the S1 subunit have also been developed and tested by *in vitro* and *in vivo* studies, such as CR3022, F26G18, F26G19, m396, 1A9, and CR3014 (27–32).

A recent study suggested the involvement of similar mechanisms of host entry in infection with SARS-CoV-2, and consequently, different studies are currently investigating single MAbs or combinations of different MAbs. Such antibodies recognize different epitopes on the SARS-CoV-2 surface, which should be assessed first by *in vitro* and *in vivo* (mouse) approaches prior to different clinical trials. However, several neutralizing MAbs also bind to IgG Fc receptors (FcγR). The antibody/FcγR interaction might lead to virus entry that could infect other cells expressing this receptor independently of the ACE2-specific virus receptor. Recently, it has been demonstrated that FcγRIIA plays a major role in viral entry via antibody-dependent enhancement (ADE) using *in vitro* strategies (46). However, the signaling pathway associated with the MAbs/virus/receptor interaction is not yet clear. ADE viral entry in the presence of neutralizing MAbs has been demonstrated for many viruses, especially for those expressing the coronavirus spike protein. Understanding the effect of this interaction on the activation of human cells expressing the Fc receptor and viral proliferation may help to establish new vaccination strategies in the future.

### Treatment of Inflammatory Cytokine Storm

#### MAbs Against the IL-6 Receptor

To explore the pathophysiological mechanisms and development of novel therapeutic approaches for sepsis, a recent study using caecal ligation and puncture (CLP) was performed in a septic mouse model. The mouse models demonstrated classical inflammatory symptoms associated with an increase in soluble triggering receptors expressed on immune cells, including interleukin (IL)-6, IL-10, TNF-α, macrophage inflammatory protein (MIP)-1α, MIP-1β, and MIP-2. These results were similar to those found in human patients with sepsis (47). IL-6 plays an important role in host defense during infections. However, exacerbation of IL-6 production favors acute severe systemic inflammation, which is named 'cytokine storm' (48). During the COVID-19 pandemic, a recent study explored the levels of cytokines, including IL-6, and the T cell frequency in three groups of individuals: healthy individuals and patients with moderate and severe COVID-19 cases. The moderate cases presented an increase in IL-6 and a decrease in the total T lymphocyte frequency. However, the severe COVID-19 cases showed an increase in IL-6, IL-2R, IL-10, and TNFα secretion associated with a severe decrease in T cells, particularly CD4+ T cells (49). These results suggest that IL-6 plays a key role in the amplification of inflammation associated with lung injury, leading to respiratory distress (37, 38). Moreover, this antibody has been used in the treatment of rheumatoid arthritis and was approved by the FDA 10 years ago, and the side effects have been extensively studied (50). Taken together, these findings suggest that IL-6 or its receptor present a potent target of interest for the treatment of COVID-19-associated acute respiratory distress syndrome (ARDS). In this context, treatment of one case of COVID-19 associated with respiratory failure with an anti-interleukin-6 receptor inhibitor named tocilizumab resulted in favorable recovery (51). To explore whether tocilizumab can be used as a treatment for COVID-19, clinical trials with a large number of patients with the correct groups should be conducted robustly to prevent mortality. However, the optimal disease stage for the administration of tocilizumab must be defined carefully. Since it has been shown that IL-6 can either suppress or facilitate viral replication (52), one crucial issue to address will be the optimal timing of anti-IL6 administration. If it occurs too early, the drugs may affect viral clearance. If it occurs too late, the drugs may not be effective. The optimal timing of the administration of anti-IL-6 must be assessed in trials. Several randomized controlled trials of tocilizumab, sarilumab and siltuximab, either alone or in combination, are now being proposed in patients with severe COVID-19 and are underway mainly in China, Western Europe, USA, Russia, Malaysia, and Australia (53). Moreover, different clinical trials are under way to evaluate the safety and efficacy of IL-6 inhibitors with various protocols and comparators. The identifiers of the clinical trials are NCT04332913, NCT04335071, NCT04317092, NCT04324073, NCT04320615, NCT04306705, NCT04315298, NCT04315480, NCT04321993, NCT04348500, NCT04329650, NCT04330638, NCT04345289, NCT04327388, NCT04341870, and NCT04322773 (ClinicalTrials.gov).

#### MAbs Against Chemokine Receptors

Several clinical trials are also ongoing to examine the effect of blocking other proinflammatory cytokines, such as TNF (54) and granulocyte–macrophage colony-stimulating factor (GM–CSF), with the clinical trial identifier NCT04341116. The aim of this study is to interfere with cytokine signaling, leading to decreased hyperinflammation in patients with severe COVID-19. Indeed, the most highly pathological macrophages are derived from the circulating monocytes infiltrating the lung. Moreover, CCR2 plays a central role in the recruitment and accumulation of monocytes in inflamed tissues (55). Altogether, these results suggest that CCR2 blockade could potentially help to reduce the accumulation of pathological monocytes in inflamed tissues. A new clinical trial (NCT04343651) targeting CCR5, another chemokine receptor that regulates monocyte and T cell recruitment, is ongoing in patients with COVID-19 with mild-to-moderate symptoms of respiratory illness.

### Chloroquine

Chloroquine (CQ) or hydroxychloroquine (HCQ) (a more soluble and less toxic metabolite of CQ) are antimalarial products that have been tested in humans (56). CQ and HCQ are also used in the treatment of several autoimmune diseases, such as systemic lupus erythematosus and rheumatoid arthritis. Additionally, CQ inhibits autophagy, favoring the apoptosis of cancer cells (57).

Promising studies have demonstrated that the CQ/HCQ compounds have the ability to inhibit certain coronaviruses, such as SARS-CoV-1 (58). Additional *in vitro* studies have shown that CQ and HCQ have antiviral activity against SARS-CoV-2, with more side effects being observed for CQ than for HCQ (58). In contrast, others have demonstrated that HCQ has greater antiviral activity than CQ during SARS-CoV-2 infection (18). Basically, CQ or HCQ exert their effects on eukaryotic cells by increasing the vacuolar pH of organelles such as endosomes and lysosomes. The increase in pH neutralizes the acidic lysosomal pH, decreasing autophagosome-lysosome fusion and autophagic degradation (59, 60). Autophagosome-lysosome fusion is essential for virus/cell fusion and immune-modulating activity (61). CQ and HCQ can also modify the glycosylation of ACE2, which binds to the spike protein S of SARS-CoV. This may interfere with the virus-receptor interaction (19). Additionally, an *in vitro* approach demonstrated that CQ inhibits COVID-19 virus infection (62). Some studies have indicated that HCQ also reduces the levels of some proinflammatory cytokines, such as IL-6, IL-18, and TNF-α (63). Indeed, CQ and HCQ inhibit endosomal TLRs and have anti-inflammatory effects by inhibiting prostaglandin synthesis or lipid peroxidation (64).

Hence, it was suggested that CQ and HCQ represent a potential new drug treatment for COVID-19. However, there are some limitations in performing clinical trials in patients owing to the restrictions on research studies using cell culture or animals and side effects, such as cardiotoxicity and liver cytotoxicity, due to the half-life of these compounds of ~3.1 days (65). However, the risk of toxicity in patients treated for 10 years with HCQ for systemic lupus erythematosus was shown to be approximately 7.5% and to be higher in patients treated for longer periods (66). In COVID-19-associated acute infection, CQ and HCQ are used for a very short time (~5 days). Nevertheless, acute adverse events, such as hypersensitivity and gastrointestinal intolerance, require attention, especially in critically ill patients who may develop similar clinical manifestations due to COVID-19. Additionally, CQ and HCQ can be safely used during pregnancy (67). Recently, a clinical trial with a small sample size showed that HCQ treatment is associated with a decrease in viral load in COVID-19 patients, and the effect is reinforced by azithromycin (39). Because of the low number of patients and the lack of some group controls during this recent study, new national and international clinical trials are being conducted to confirm the authenticity of these findings. A current clinical trial of CQ and HCQ therapy in the treatment of COVID-19 in Europe may reveal new possibilities for antiviral therapy for COVID-19 to stop the pandemic. Although the antiviral activity of hydroxychloroquine remains uncertain, there have been several controversies regarding the clinical benefits of this drug in patients with COVID-19. Recently, a new publication showed the beneficial effects of hydroxychloroquine or chloroquine when used alone or with a macrolide on in-hospital outcomes for COVID-19. Each of the drug regimens was associated with a decrease in in-hospital survival and an increased frequency of ventricular arrhythmias when used for the treatment of COVID-19. However, this study was retracted from the Lancet journal (https://doi.org/10.1016/S0140-6736(20)31174-0). In contrast, an approved study by the Ethics Committee of Shanghai Public Health Clinical Center under the number NCT04261517 demonstrated that the prognosis of COVID-19 patients with moderate cases is good. However, a large sample size study is needed to investigate the effects of HCQ in the treatment of COVID-19 (68). A new study is ongoing and can be found on ClinicalTrials.gov under the identifier NCT04303507 with the official title “Chloroquine/Hydroxychloroquine Prevention of Coronavirus Disease (COVID-19).” This study is a double-blinded, randomized, and placebo-controlled trial that will be conducted in a healthcare setting. A total of 40,000 participants will be recruited, and the investigators predict an average of 400–800 participants per site at 50–100 sites. However, the estimated completion date is April 2021.

### Anticoagulant Treatments

Since recent findings revealed that most COVID-19 patients with severe cases admitted to the intensive care unit for respiratory failure present predominantly with hypercoagulation, anticoagulant drugs could a potentially prevent a state that could lead to arterial and venous thromboembolic complications (69). Antithrombin and activated protein C for the treatment of classical acute respiratory distress syndrome can be used as anticoagulants for inflammatory thrombus prevention. Platelets may be involved in systemic and local thrombotic responses. Antiplatelet therapies may present a new therapeutic approach. This is a known phenomenon in acute coronary syndromes, where anticoagulant therapy along with antiplatelet therapy decreases arterial thrombosis, but it is associated with an increase in bleeding risk (42).

### Therapies Targeting Viral Replication

Remdesivir is an antiviral molecule with a chemical formula of C27H35N6O8P. Remdesivir prevents viral replication by inhibiting viral DNA polymerase. Its antiviral activity has been demonstrated against Ebola virus in multiple human cell types, including primary macrophages and human endothelial cells, with low half-maximal effective concentration (EC_50_) values of 0.06–0.14 μM (33). It has also been shown that remdesivir inhibits SARS-CoV in primary human airway epithelial cell cultures, which are a biologically relevant *in vitro* model of pulmonary infection (70). Moreover, remdesivir has exhibited antiviral activity against the Marburg virus (33). SARS-CoV and SARS-CoV-2 present 82% RNA sequence homology, and their RNA-dependent RNA polymerase (RdRp) sequences share 96% sequence similarity. Therefore, drugs targeting the viral RdRp proteins of SARS-CoV are also suspected to be effective against SARS-CoV-2. According to the *in vitro* antiviral activity of remdesivir, the *in vivo* tests showed the suppression of Ebola virus replication and the protection of all infected animals against lethal infection (33). In addition, remdesivir decreased the viral load in the lungs and preserved the pulmonary function of mice during SARS-CoV infection (70). These findings suggest that remdesivir can be used as a potential new therapeutic approach for human infections caused by coronaviruses, including SARS-CoV-2. In fact, the first case of COVID-19 in Washington, USA, was treated with intravenous remdesivir. During the treatment, no obvious adverse effects were observed (71). However, we cannot comment yet on the efficiency of the treatment effect of remdesivir during the COVID-19 outbreak.

There are four clinical trials currently enrolling patients in the United States. Moreover, two clinical trials in China have been registered on ClinicalTrials.gov: NCT04257656 for severe disease and NCT04252664 for mild-to-moderate disease (72). Recently, Yeming et al. published the results of the NCT04257656 clinical trial, which showed no clear outcome because of the death or discharge of patients (73). Moreover, in another clinical trial, the benefit in terms of the time to clinical improvement was not statistically significant (21 vs. 23 days), even though the study was underpowered (74). There are limited safety data for remdesivir, which should be obtained in further studies.

### Therapies Targeting Viral Transcription

Ribavirin is a broad-spectrum nucleoside antiviral drug that is phosphorylated in virus-infected cells. Basically, the entry of the product into virus-infected cells leads to its phosphorylation. This product acts as a competitive inhibitor of the viral synthetase, interfering with early viral transcription events and thereby hindering the synthesis of ribonucleoproteins and subsequent viral spread. Several controversial *in vitro* studies investigating ribavirin have been conducted. While a few of them have demonstrated that ribavirin has an antiviral effect on SARS, others have revealed no evidence of its antiviral role (75, 76). Additionally, a clinical trial reported no significant antiviral effects on SARS-infected patients (77). In fact, the same study reported side effects, such as haemolytic anemia, resulting from the clinical administration of ribavirin (77). During the COVID-19 pandemic, ribavirin combined with interferon was used based on the Chinese treatment guidelines.

### BCG Vaccine

The Bacillus Calmette-Guerin (BCG) vaccine against tuberculosis has been demonstrated to reduce mortality during other infections. The protective mechanism involved in tuberculosis infection has been explored *in vivo*. It was demonstrated that BCG vaccination increased IFN-γ production by CD4+ cells (78). T cells play a crucial role in viral infections; CD4 T cells provide B cell help for antibody production and control the response of other immune cell subsets, whereas CD8 T cells kill infected cells to reduce the viral burden. To better understand the role of T cell responses in SARS-CoV-2 infection, some studies are beginning to be conducted. During SARS-CoV-1 infection, the occurrence of lymphopenia with drastically reduced numbers of both CD4 and CD8 T cells in moderate and severe COVID-19 cases has been described in several current reports (79). Th1 and Th17 cells play a crucial role in the induction of CD4+ and CD8+ memory cells that are involved in the control of the immune system response during non-mycobacterial secondary infections. Interestingly, BCG vaccination continued to increase Th1 and Th17 responses at least 1 year after vaccination in healthy subjects (80). COVID-19 infection severity is associated with a sharp decrease in the frequency of CD4+ and CD8+ cells and the expression of INF-γ on the surface of CD4+ cells (78). The nonspecific effects of the BCG vaccine present a potential therapeutic method to increase memory responses and enhance the immune system during viral infections that might aid in combating the COVID-19 pandemic.

## Discussion

Improved understanding of the viral entry mechanisms and the inflammatory response generated during infection would allow the development of appropriate therapeutic strategies to manage patients with COVID-19. The different therapeutic strategies (Table 1) discussed in this review are encouraging and have been proposed to treat or prevent the spread of COVID-19. In addition, most of the described compounds are readily available, and they are known to result in a minor risk of adverse events. Several clinical trials are in process to validate the results. However, these strategies are not without risks, and special attention to factors such as age, sex, and associations with other chronic diseases must be considered during patient selection. Non-specific proinflammatory cytokine targeting during COVID-19 treatment using corticoids, e.g., may favor viral spread. However, targeting specific individual cytokines does not increase viral infection and prevents cytokine storm inflammation-mediated tissue injury, notably in the lung. Since observations have indicated that there are two stages of disease, the first of which is characterized by virus spread and the second by the hyperproinflammatory response responsible for respiratory distress, the timing of the initiation of therapy needs to be carefully defined.

In this review, we also mentioned that virus-neutralizing MAbs represent a therapeutic method with a high potential to prevent viral spread. However, the use of immunoglobulin class G (IgG) MAbs may contribute to an ADE mechanism favoring the spread of the virus during treatment. In parallel, these antibodies can also induce anaphylactic shock that is mediated by the FcγR receptor; FcγRIIA is expressed by neutrophils and platelets, in particular (81). These side effects remain poorly studied. The development of IgG4 or F(ab)′_2_ antibodies to neutralize the virus or to target proinflammatory antibodies that cannot interact with FcγR may prevent this risk.

Finally, the treatment duration should be well-defined in terms of the half-life of molecules to prevent liver toxicity and the immunosuppressive effect.

Thus, the use of monotherapy or combinatorial therapeutic strategies during different stages of COVID-19 infection represent a potential therapeutic strategy to stop the ongoing pandemic.

## Author Contributions

SB and AB wrote this review. Both authors contributed to the article and approved the submitted version.

## Conflict of Interest

The authors declare that the research was conducted in the absence of any commercial or financial relationships that could be construed as a potential conflict of interest.

## References

[1] Corman VM Lienau J Witzenrath M [Coronaviruses as the cause of respiratory infections]. Internist (Berl). (2019) 60:1136–45. 10.1007/s00108-019-00671-5 31455974 PMC7079972

[2] Fehr AR Channappanavar R Perlman S. Middle east respiratory syndrome: emergence of a pathogenic human coronavirus. Annu Rev Med. (2017) 68:387–99. 10.1146/annurev-med-051215-031152 27576010 PMC5353356

[3] Hoffmann M Kleine-Weber H Schroeder S Kruger N Herrler T Erichsen S. SARS-CoV-2 cell entry depends on ACE2 and TMPRSS2 and is blocked by a clinically proven protease inhibitor. Cell. (2020) 181:271–80.e8. 10.1016/j.cell.2020.02.052 32142651 PMC7102627

[4] Lau JT Yang X Pang E Tsui HY Wong E Wing YK. SARS-related perceptions in Hong Kong. Emerg Infect Dis. (2005) 11:417–24. 10.3201/eid1103.040675 15757557 PMC3298267

[5] Guan Y Zheng BJ He YQ Liu XL Zhuang ZX Cheung CL. Isolation and characterization of viruses related to the SARS coronavirus from animals in southern China. Science. (2003) 302:276–8. 10.1126/science.1087139 12958366

[6] Huang C Wang Y Li X Ren L Zhao J Hu Y. Clinical features of patients infected with 2019 novel coronavirus in Wuhan, China. Lancet. (2020) 395:497–506. 10.1016/S0140-6736(20)30183-5 31986264 PMC7159299

[7] Chan JF Yuan S Kok KH To KK Chu H Yang J. A familial cluster of pneumonia associated with the 2019 novel coronavirus indicating person-to-person transmission: a study of a family cluster. Lancet. (2020) 395:514–23. 10.1016/S0140-6736(20)30154-9 31986261 PMC7159286

[8] Zhu F Cao Y Xu S Zhou M Reply to Comments on 'Co-infection of SARS-CoV-2 and HIV in a patient in Wuhan city, China'. J Med Virol. (2020) 10.1002/jmv.25838. [Epub ahead of print]. PMC7228399 32160316

[9] Matsuyama S Nagata N Shirato K Kawase M Takeda M Taguchi F. Efficient activation of the severe acute respiratory syndrome coronavirus spike protein by the transmembrane protease TMPRSS2. J Virol. (2010) 84:12658–64. 10.1128/JVI.01542-10 20926566 PMC3004351

[10] Shulla A Heald-Sargent T Subramanya G Zhao J Perlman S Gallagher T. A transmembrane serine protease is linked to the severe acute respiratory syndrome coronavirus receptor and activates virus entry. J Virol. (2011) 85:873–82. 10.1128/JVI.02062-10 21068237 PMC3020023

[11] Imai Y Kuba K Penninger JM. Angiotensin-converting enzyme 2 in acute respiratory distress syndrome. Cell Mol Life Sci. (2007) 64:2006–12. 10.1007/s00018-007-6228-6 17558469 PMC7079778

[12] Skeggs LT Dorer FE Levine M Lentz KE Kahn JR. The biochemistry of the renin-angiotensin system. Adv Exp Med Biol. (1980) 130:1–27. 10.1007/978-1-4615-9173-3_1 6250339

[13] Corvol P Williams TA Soubrier F. Peptidyl dipeptidase a: angiotensin I-converting enzyme. Methods Enzymol. (1995) 248:283–305. 10.1016/0076-6879(95)48020-X 7674927

[14] Tschope C Schultheiss HP Walther T. Multiple interactions between the renin-angiotensin and the kallikrein-kinin systems: role of ACE inhibition and AT1 receptor blockade. J Cardiovasc Pharmacol. (2002) 39:478–87. 10.1097/00005344-200204000-00003 11904521

[15] Li F Li W Farzan M Harrison SC. Structure of SARS coronavirus spike receptor-binding domain complexed with receptor. Science. (2005) 309:1864–8. 10.1126/science.1116480 16166518

[16] Gu Y Hsu AC Pang Z Pan H Zuo X Wang G. Role of the innate cytokine storm induced by the influenza A virus. Viral Immunol. (2019) 32:244–51. 10.1089/vim.2019.0032 31188076

[17] England JT Abdulla A Biggs CM Lee AYY Hay KA Hoiland RL. Weathering the COVID-19 storm: lessons from hematologic cytokine syndromes. Blood Rev. (2020) 2020:100707. 10.1016/j.blre.2020.100707 32425294 PMC7227559

[18] Yao X Ye F Zhang M Cui C Huang B Niu P. In vitro antiviral activity and projection of optimized dosing design of hydroxychloroquine for the treatment of severe acute respiratory syndrome coronavirus 2 (SARS-CoV-2). Clin Infect Dis. (2020) ciaa237. 10.1093/cid/ciaa237. [Epub ahead of print]. 32150618 PMC7108130

[19] Wang M Cao R Zhang L Yang X Liu J Xu M. Remdesivir and chloroquine effectively inhibit the recently emerged novel coronavirus (2019-nCoV) in vitro. Cell Res. (2020) 30:269–71. 10.1038/s41422-020-0282-0 32020029 PMC7054408

[20] Wu R Wang L Kuo HD Shannar A Peter R Chou PJ. An update on current therapeutic drugs treating COVID-19. Curr Pharmacol Rep. (2020) 2020:1–15. 10.1007/s40495-020-00216-7 32395418 PMC7211915

[21] Silva JC Mariz HA Rocha LF Jr Oliveira PS Dantas AT. Hydroxychloroquine decreases Th17-related cytokines in systemic lupus erythematosus and rheumatoid arthritis patients. Clinics (São Paulo). (2013) 68:766–71. 10.6061/clinics/2013(06)07 23778483 PMC3674253

[22] Ohkoshi M Fujii S Effect of the synthetic protease inhibitor [N,N-dimethylcarbamoyl-methyl 4-(4-guanidinobenzoyloxy)-phenylacetate] methanesulfate on carcinogenesis by 3-methylcholanthrene in mouse skin. J Natl Cancer Inst. (1983) 71:1053–7. 6580482

[23] Ikeda S Manabe M Muramatsu T Takamori K Ogawa H. Protease inhibitor therapy for recessive dystrophic epidermolysis bullosa. In vitro effect and clinical trial with camostat mesylate. J Am Acad Dermatol. (1988) 18:1246–52. 10.1016/S0190-9622(88)70130-9 3385039

[24] Sai JK Suyama M Kubokawa Y Matsumura Y Inami K Watanabe S. Efficacy of camostat mesilate against dyspepsia associated with non-alcoholic mild pancreatic disease. J Gastroenterol. (2010) 45:335–41. 10.1007/s00535-009-0148-1 19876587

[25] Yamamoto M Matsuyama S Li X Takeda M Kawaguchi Y Inoue JI. Identification of nafamostat as a potent inhibitor of middle east respiratory syndrome coronavirus s protein-mediated membrane fusion using the split-protein-based cell-cell fusion assay. Antimicrob Agents Chemother. (2016) 60:6532–9. 10.1128/AAC.01043-16 27550352 PMC5075056

[26] Iwaki M Ino Y Motoyoshi A Ozeki M Sato T Kurumi M. Pharmacological studies of FUT-175, nafamostat mesilate. V. Effects on the pancreatic enzymes and experimental acute pancreatitis in rats. Jpn J Pharmacol. (1986) 41:155–62. 10.1254/jjp.41.155 2427760

[27] Berry JD Hay K Rini JM Yu M Wang L Plummer FA Neutralizing epitopes of the SARS-CoV S-protein cluster independent of repertoire, antigen structure or mAb technology. MAbs. (2010) 2:53–66. 10.4161/mabs.2.1.10788 20168090 PMC2828578

[28] ter Meulen J van den Brink EN Poon LL Marissen WE Leung CS Cox F. Human monoclonal antibody combination against SARS coronavirus: synergy and coverage of escape mutants. PLoS Med. (2006) 3:e237. 10.1371/journal.pmed.0030237 16796401 PMC1483912

[29] Ng OW Keng CT Leung CS Peiris JS Poon LL Tan YJ. Substitution at aspartic acid 1128 in the SARS coronavirus spike glycoprotein mediates escape from a S2 domain-targeting neutralizing monoclonal antibody. PLoS ONE. (2014) 9:e102415. 10.1371/journal.pone.0102415 25019613 PMC4097068

[30] Lip KM Shen S Yang X Keng CT Zhang A Oh HL Monoclonal antibodies targeting the HR2 domain and the region immediately upstream of the HR2 of the S protein neutralize in vitro infection of severe acute respiratory syndrome coronavirus. J Virol. (2006) 80:941–50. 10.1128/JVI.80.2.941-950.2006 16378996 PMC1346840

[31] ter Meulen J Bakker AB van den Brink EN Weverling GJ Martina BE Haagmans BL. Human monoclonal antibody as prophylaxis for SARS coronavirus infection in ferrets. Lancet. (2004) 363:2139–41. 10.1016/S0140-6736(04)16506-9 15220038 PMC7112500

[32] van den Brink EN Ter Meulen J Cox F Jongeneelen MA Thijsse A Throsby M. Molecular and biological characterization of human monoclonal antibodies binding to the spike and nucleocapsid proteins of severe acute respiratory syndrome coronavirus. J Virol. (2005) 79:1635–44. 10.1128/JVI.79.3.1635-1644.2005 15650189 PMC544131

[33] Warren TK Jordan R Lo MK Ray AS Mackman RL Soloveva V. Therapeutic efficacy of the small molecule GS-5734 against Ebola virus in rhesus monkeys. Nature. (2016) 531:381–5. 10.1038/nature17180 26934220 PMC5551389

[34] Agostini ML Andres EL Sims AC Graham RL Sheahan TP Lu X. Coronavirus susceptibility to the antiviral remdesivir (GS-5734) is mediated by the viral polymerase and the proofreading exoribonuclease. mBio. (2018) 9:e00221-18. 10.1128/mBio.00221-18 29511076 PMC5844999

[35] Lim J Jeon S Shin HY Kim MJ Seong YM Lee WJ Case of the index patient who caused tertiary transmission of COVID-19 infection in korea: the application of lopinavir/ritonavir for the treatment of COVID-19 infected pneumonia monitored by quantitative RT-PCR. J Korean Med Sci. (2020) 35:e79 10.3346/jkms.2020.35.e89 32056407 PMC7025910

[36] Pecheur EI Borisevich V Halfmann P Morrey JD Smee DF Prichard M. The synthetic antiviral drug arbidol inhibits globally prevalent pathogenic viruses. J Virol. (2016) 90:3086–92. 10.1128/JVI.02077-15 26739045 PMC4810626

[37] Conti P Ronconi G Caraffa A Gallenga CE Ross R Frydas I. Induction of pro-inflammatory cytokines (IL-1 and IL-6) and lung inflammation by Coronavirus-19 (COVI-19 or SARS-CoV-2): anti-inflammatory strategies. J Biol Regul Homeost Agents. (2020) 34:1. 10.23812/CONTI-E 32171193

[38] Chen N Zhou M Dong X Qu J Gong F Han Y. Epidemiological and clinical characteristics of 99 cases of 2019 novel coronavirus pneumonia in Wuhan, China: a descriptive study. Lancet. (2020) 395:507–13. 10.1016/S0140-6736(20)30211-7 32007143 PMC7135076

[39] Gautret P Lagier JC Parola P Hoang VT Meddeb L Mailhe M Hydroxychloroquine and azithromycin as a treatment of COVID-19: results of an open-label non-randomized clinical trial. Int J Antimicrob Agents. (2020) 2020:105949 10.1016/j.ijantimicag.2020.105949 PMC7102549 32205204

[40] Molina JM Delaugerre C Le Goff J Mela-Lima B Ponscarme D Goldwirt L No evidence of rapid antiviral clearance or clinical benefit with the combination of hydroxychloroquine and azithromycin in patients with severe COVID-19 infection. Med Mal Infect. (2020) 50:384 10.1016/j.medmal.2020.03.006 32240719 PMC7195369

[41] Russell CD Millar JE Baillie JK. Clinical evidence does not support corticosteroid treatment for 2019-nCoV lung injury. Lancet. (2020) 395:473–5. 10.1016/S0140-6736(20)30317-2 32043983 PMC7134694

[42] Iba T Levy JH Levi M Connors JM Thachil J Coagulopathy of coronavirus disease 2019. Crit Care Med. (2020) 10.1097/CCM.0000000000004458 PMC7255402 32467443

[43] Liu Y Sun W Guo Y Chen L Zhang L Zhao S. Association between platelet parameters and mortality in coronavirus disease 2019: retrospective cohort study. Platelets. (2020) 31:490–6. 10.1080/09537104.2020.1754383 32297540 PMC7171387

[44] Shanmugaraj B Siriwattananon K Wangkanont K Phoolcharoen W. Perspectives on monoclonal antibody therapy as potential therapeutic intervention for Coronavirus disease-19 (COVID-19). Asian Pac J Allergy Immunol. (2020) 38:10–8. 10.12932/AP-200220-0773 32134278

[45] Sui J Li W Roberts A Matthews LJ Murakami A Vogel L. Evaluation of human monoclonal antibody 80R for immunoprophylaxis of severe acute respiratory syndrome by an animal study, epitope mapping, and analysis of spike variants. J Virol. (2005) 79:5900–6. 10.1128/JVI.79.10.5900-5906.2005 15857975 PMC1091676

[46] Wan Y Shang J Graham R Baric RS Li F. Receptor recognition by the novel coronavirus from wuhan: an analysis based on decade-long structural studies of SARS coronavirus. J Virol. (2020) 94:e00127-20. 10.1128/JVI.00127-20 31996437 PMC7081895

[47] Li JL Li G Jing XZ Li YF Ye QY Jia HH. Assessment of clinical sepsis-associated biomarkers in a septic mouse model. J Int Med Res. (2018) 46:2410–22. 10.1177/0300060518764717 29644918 PMC6023044

[48] Tanaka T Narazaki M Kishimoto T. Immunotherapeutic implications of IL-6 blockade for cytokine storm. Immunotherapy. (2016) 8:959–70. 10.2217/imt-2016-0020 27381687

[49] Pedersen SF Ho YC. SARS-CoV-2: a storm is raging. J Clin Invest. (2020) 130:2202–5. 10.1172/JCI137647 32217834 PMC7190904

[50] Izumiyama T Mori Y Mori S Mori N Kodama T Itoi E. The effect of anti-IL-6 receptor antibody for the treatment of McH-lpr/lpr-RA1 mice that spontaneously developed destructive arthritis and enthesitis. BMC Musculoskelet Disord. (2019) 20:286. 10.1186/s12891-019-2664-3 31200688 PMC6570918

[51] Michot JM Albiges L Chaput N Saada V Pommeret F Griscelli F. Tocilizumab, an anti-IL6 receptor antibody, to treat Covid-19-related respiratory failure: a case report. Ann Oncol. (2020) 31:961–4. 10.1016/j.annonc.2020.03.300 32247642 PMC7136869

[52] Velazquez-Salinas L Verdugo-Rodriguez A Rodriguez LL Borca MV. The role of interleukin 6 during viral infections. Front Microbiol. (2019) 10:1057. 10.3389/fmicb.2019.01057 31134045 PMC6524401

[53] Atal S Fatima Z. IL-6 inhibitors in the treatment of serious COVID-19: a promising therapy? Pharmaceut Med. (2020) 1–9. 10.1007/s40290-020-00342-z. [Epub ahead of print]. 32535732 PMC7292936

[54] Feldmann M Maini RN Woody JN Holgate ST Winter G Rowland M. Trials of anti-tumour necrosis factor therapy for COVID-19 are urgently needed. Lancet. (2020) 395:1407–9. 10.1016/S0140-6736(20)30858-8 32278362 PMC7158940

[55] Serbina NV Pamer EG. Monocyte emigration from bone marrow during bacterial infection requires signals mediated by chemokine receptor CCR2. Nat Immunol. (2006) 7:311–7. 10.1038/ni1309 16462739

[56] Krafts K Hempelmann E Skorska-Stania A. From methylene blue to chloroquine: a brief review of the development of an antimalarial therapy. Parasitol Res. (2012) 111:1–6. 10.1007/s00436-012-2886-x 22411634

[57] Lamoureux F Zoubeidi A. Dual inhibition of autophagy and the AKT pathway in prostate cancer. Autophagy. (2013) 9:1119–20. 10.4161/auto.24921 23670050 PMC3722327

[58] Li G De Clercq E. Therapeutic options for the 2019 novel coronavirus (2019-nCoV). Nat Rev Drug Discov. (2020) 19:149–50. 10.1038/d41573-020-00016-0 32127666

[59] Mizushima N Yoshimori T Levine B. Methods in mammalian autophagy research. Cell. (2010) 140:313–26. 10.1016/j.cell.2010.01.028 20144757 PMC2852113

[60] Solomon VR Lee H. Chloroquine and its analogs: a new promise of an old drug for effective and safe cancer therapies. Eur J Pharmacol. (2009) 625:220–33. 10.1016/j.ejphar.2009.06.063 19836374

[61] Savarino A Di Trani L Donatelli I Cauda R Cassone A. New insights into the antiviral effects of chloroquine. Lancet Infect Dis. (2006) 6:67–9. 10.1016/S1473-3099(06)70361-9 16439323 PMC7129107

[62] Vincent MJ Bergeron E Benjannet S Erickson BR Rollin PE Ksiazek TG. Chloroquine is a potent inhibitor of SARS coronavirus infection and spread. Virol J. (2005) 2:69. 10.1186/1743-422X-2-69 16115318 PMC1232869

[63] van den Borne BE Dijkmans BA de Rooij HH le Cessie S Verweij CL. Chloroquine and hydroxychloroquine equally affect tumor necrosis factor-alpha, interleukin 6, and interferon-gamma production by peripheral blood mononuclear cells. J Rheumatol. (1997) 24:55–60. 9002011

[64] Kuznik A Bencina M Svajger U Jeras M Rozman B Jerala R. Mechanism of endosomal TLR inhibition by antimalarial drugs and imidazoquinolines. J Immunol. (2011) 186:4794–804. 10.4049/jimmunol.1000702 21398612

[65] Coelho AS Chagas CEP de Padua RM Pianetti GA Fernandes C. A comprehensive stability-indicating HPLC method for determination of chloroquine in active pharmaceutical ingredient and tablets: Identification of oxidation impurities. J Pharm Biomed Anal. (2017) 145:248–54. 10.1016/j.jpba.2017.06.023 28668653

[66] Melles RB Marmor MF. The risk of toxic retinopathy in patients on long-term hydroxychloroquine therapy. JAMA Ophthalmol. (2014) 132:1453–60. 10.1001/jamaophthalmol.2014.3459 25275721

[67] Zhao X Jiang Y Zhao Y Xi H Liu C Qu F. Analysis of the susceptibility to COVID-19 in pregnancy and recommendations on potential drug screening. Eur J Clin Microbiol Infect Dis. (2020) 39:1209–20. 10.1007/s10096-020-03897-6 32328850 PMC7178925

[68] Chen J Liu D Liu L Liu P Xu Q Xia L. [A pilot study of hydroxychloroquine in treatment of patients with moderate COVID-19]. Zhejiang Da Xue Xue Bao Yi Xue Ban. (2020) 49:215–9. 32391667 10.3785/j.issn.1008-9292.2020.03.03 PMC8800713

[69] Spiezia L Boscolo A Poletto F Cerruti L Tiberio I Campello E. COVID-19-related severe hypercoagulability in patients admitted to intensive care unit for acute respiratory failure. Thromb Haemost. (2020) 120:998–1000. 10.1055/s-0040-1710018 32316063 PMC7295272

[70] Sheahan TP Sims AC Graham RL Menachery VD Gralinski LE Case JB Broad-spectrum antiviral GS-5734 inhibits both epidemic and zoonotic coronaviruses. Sci Transl Med. (2017) 9:396 10.1126/scitranslmed.aal3653 PMC5567817 28659436

[71] Ko WC Rolain JM Lee NY Chen PL Huang CT Lee PI. Arguments in favour of remdesivir for treating SARS-CoV-2 infections. Int J Antimicrob Agents. (2020) 2020:105933. 10.1016/j.ijantimicag.2020.105933 32147516 PMC7135364

[72] Bhimraj A Morgan RL Shumaker AH Lavergne V Baden L Cheng VC. Infectious diseases society of america guidelines on the treatment and management of patients with COVID-19. Clin Infect Dis. (2020) 10.1093/cid/ciaa478. [Epub ahead of print]. 32338708 PMC7197612

[73] Wang Y Zhou F Zhang D Zhao J Du R Hu Y. Evaluation of the efficacy and safety of intravenous remdesivir in adult patients with severe COVID-19: study protocol for a phase 3 randomized, double-blind, placebo-controlled, multicentre trial. Trials. (2020) 21:422. 10.1186/s13063-020-04352-9 32448345 PMC7245636

[74] Davies M Osborne V Lane S Roy D Dhanda S Evans A. Remdesivir in treatment of COVID-19: a systematic benefit-risk assessment. Drug Saf. (2020) 43:645–56. 10.1007/s40264-020-00966-9 32468196 PMC7255634

[75] Chen F Chan KH Jiang Y Kao RY Lu HT Fan KW. In vitro susceptibility of 10 clinical isolates of SARS coronavirus to selected antiviral compounds. J Clin Virol. (2004) 31:69–75. 10.1016/j.jcv.2004.03.003 15288617 PMC7128415

[76] Morgenstern B Michaelis M Baer PC Doerr HW Cinatl J Jr. Ribavirin and interferon-beta synergistically inhibit SARS-associated coronavirus replication in animal and human cell lines. Biochem Biophys Res Commun. (2005). 326:905–8. 10.1016/j.bbrc.2004.11.128 15607755 PMC7092851

[77] Booth CM Matukas LM Tomlinson GA Rachlis AR Rose DB Dwosh HA. Clinical features and short-term outcomes of 144 patients with SARS in the greater Toronto area. JAMA. (2003) 289:2801–9. 10.1001/jama.289.21.JOC30885 12734147

[78] Mathurin KS Martens GW Kornfeld H Welsh RM. CD4 T-cell-mediated heterologous immunity between mycobacteria and poxviruses. J Virol. (2009) 83:3528–39. 10.1128/JVI.02393-08 19193795 PMC2663272

[79] Diao B Wang C Tan Y Chen X Liu Y Ning L. Reduction and functional exhaustion of t cells in patients with coronavirus disease 2019 (COVID-19). Front Immunol. (2020). 11:827. 10.3389/fimmu.2020.00827 32425950 PMC7205903

[80] Kleinnijenhuis J Quintin J Preijers F Joosten LA Jacobs C Xavier RJ. BCG-induced trained immunity in NK cells: Role for non-specific protection to infection. Clin Immunol. (2014) 155:213–9. 10.1016/j.clim.2014.10.005 25451159 PMC5084088

[81] Jonsson F de Chaisemartin L Granger V Gouel-Cheron A Gillis CM Zhu Q. An IgG-induced neutrophil activation pathway contributes to human drug-induced anaphylaxis. Sci Transl Med. (2019) 11:eaat1479. 10.1126/scitranslmed.aat1479 31292264

